# Efficacy and Safety of Atezolizumab Plus Bevacizumab for Patients With Hepatocellular Carcinoma and Child–Pugh Class B

**DOI:** 10.1111/liv.70466

**Published:** 2025-11-28

**Authors:** Ryu Sasaki, Shigeo Shimose, Issei Saeki, Takanori Ito, Yasuto Takeuchi, Joji Tani, Tetsu Tomonari, Kyo Sasaki, Satoru Kakizaki, Takeshi Hatanaka, Satoshi Miuma, Tomotake Shirono, Hideki Iwamoto, Norikazu Tanabe, Takafumi Yamamoto, Yuki Kanayama, Atsushi Naganuma, Sohji Nishina, Tetsuji Takayama, Hideki Kobara, Motoyuki Otsuka, Hiroki Kawashima, Taro Takami, Takumi Kawaguchi, Hisamitsu Miyaaki, Hepatology InVestigator Experts in Japan (HIVE‐J) Study Group

**Affiliations:** ^1^ Department of Gastroenterology and Hepatology Nagasaki University Graduate School of Biomedical Sciences Nagasaki Japan; ^2^ Division of Gastroenterology, Department of Medicine Kurume University School of Medicine Kurume Japan; ^3^ Department of Gastroenterology and Hepatology Yamaguchi University Graduate School of Medicine Yamaguchi Japan; ^4^ Department of Gastroenterology and Hepatology Nagoya University Graduate School of Medicine Nagoya Japan; ^5^ Department of Gastroenterology Okayama University Hospital Okayama Japan; ^6^ Department of Gastroenterology and Neurology, Faculty of Medicine Kagawa University Takamatsu Kagawa Japan; ^7^ Department of Gastroenterology and Oncology Tokushima University Graduate School of Biomedical Sciences Tokushima Japan; ^8^ Department of Gastroenterology and Hepatology Kawasaki Medical School Kurashiki Japan; ^9^ Department of Clinical Research NHO Takasaki General Medical Center Takasaki Gunma Japan; ^10^ Department of Gastroenterology Gunma Saiseikai Maebashi Hospital Maebashi Japan; ^11^ Iwamoto Internal Medical Clinic Kitakyusyu Japan; ^12^ Department of Gastroenterology Toyohashi Municipal Hospital Toyohashi Japan; ^13^ Department of Gastroenterology NHO Takasaki General Medical Center Takasaki Gunma Japan

**Keywords:** atezolizumab, bevacizumab, Child–Pugh class B, hepatocellular carcinoma, modified albumin–bilirubin grade

## Abstract

**Background & Aims:**

Despite the advances in systemic therapy for unresectable hepatocellular carcinoma (HCC), patients with Child–Pugh class B (CP‐B) liver function face a significant unmet need. This study evaluated the efficacy and safety of atezolizumab plus bevacizumab (Atez/Bev) in patients with unresectable HCC and CP‐B.

**Methods:**

This retrospective study included 796 patients who received Atez/Bev between October 2020 and July 2024 from 10 institutions in Japan. The median observation period was 14.6 months. The liver function was assessed using the CP classification and modified ALBI (mALBI) grade. The progression‐free survival (PFS), overall survival (OS) and median survival time (MST) were evaluated.

**Results:**

Patients with CP‐B had significantly shorter PFS and OS than those with CP‐A (median PFS, 4.6 months vs. 7.0 months; MST, 10.3 months vs. 23.2 months) (PFS, *p* = 0.009; OS, *p* < 0.001). Although CP‐B was associated with a higher incidence of bleeding‐related events, the discontinuation rate due to adverse events did not differ from that of CP‐A. As a factor for stratifying CP‐B outcomes, significant differences in the PFS, OS and response rate were observed between mALBI grades ≤ 2b and 3 (PFS, *p* = 0.004; OS, *p* = 0.024; response rate, *p* = 0.001). In multivariate analysis, the mALBI grade (hazard ratio [95% CI]: 2.388 [1.186–4.810]; *p* = 0.014) was extracted as a factor contributing to OS in patients with CP‐B.

**Conclusion:**

Atez/Bev therapy demonstrated efficacy and safety in patients with CP‐B, especially when hepatic reserve is maintained within mALBI grade 2b.

## Lay Summary

This study shows that atezolizumab plus bevacizumab can be a safe and effective treatment option for patients with unresectable liver cancer and moderate liver impairment (Child–Pugh class B), particularly those with preserved liver function up to mALBI grade 2b. These findings may help expand treatment options for a population with limited existing choices.

**Abbreviations**
AEs: adverse events
ALBI: albumin–bilirubin
Atez/Bev: atezolizumab plus bevacizumab
BCLC: Barcelona Clinic Liver Cancer
BSC: best supportive care
CP: Child–Pugh
CP‐B: Child–Pugh class B
CPS: CP score
DC: disease control
HCC: hepatocellular carcinoma
ICI: immune checkpoint inhibitor
irAEs: immune‐related AEs
mALBI: modified ALBI
MST: median survival time
OS: overall survival
PD: progressive disease
PD‐1: programmed cell death protein 1
PD‐L1: programmed death ligand 1
PFS: progression‐free survival
PS: performance status
RECIST: Response Evaluation Criteria in Solid Tumours
VEGF: vascular endothelial growth factor

## 1 Introduction

Systemic therapy for unresectable hepatocellular carcinoma (HCC) has advanced [1, 2, 3], involving immune checkpoint inhibitor (ICI)‐based regimens for improved treatment outcomes [4, 5, 6]. Based on the results of global trials, combination therapy, including a programmed cell death protein 1 (PD‐1) inhibitor or programmed death ligand 1 (PD‐L1) inhibitor, should be considered as the first‐line standard treatment for patients without contraindications to ICI and bevacizumab. However, the benefits of ICI‐based systemic therapy for HCC have often been discussed only in patients with preserved hepatic reserves. Most clinical trials on systemic therapy for unresectable HCC exclude patients with Child–Pugh (CP) class B (CP‐B) and only include patients with CP class A (CP‐A). Data from registry studies have revealed that a certain proportion of patients with HCC have not maintained a hepatic reserve, and there is a significant unmet need for systemic therapy against HCC in patients with CP‐B liver function [7]. Nevertheless, evidence regarding the efficacy and safety of systemic therapy in patients with CP‐B liver function has not been thoroughly reviewed. Furthermore, the clinical course of patients with HCC and CP‐B liver function when ICI‐based regimens are used as the first‐line therapy is unclear. Therefore, this study evaluated the efficacy and safety of atezolizumab plus bevacizumab (Atez/Bev) for patients with unresectable HCC and CP‐B liver function in a clinical setting.

## 2 Methods

### 2.1 Patients

**FIGURE 1:**
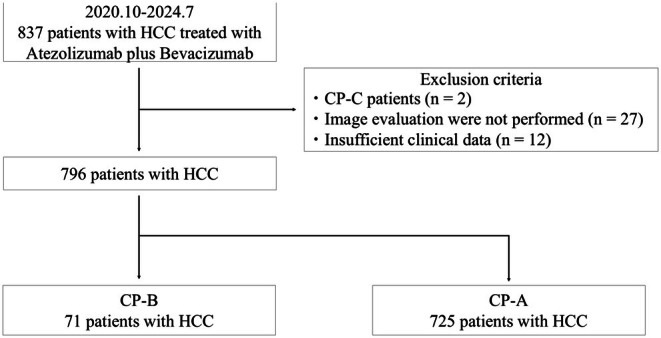
Patient selection flowchart.

This retrospective study included 837 patients who underwent Atez/Bev treatment for HCC with measurable lesions between October 2020 and July 2024. It was conducted at 10 institutions in Japan: Nagasaki University Hospital, Kurume University Hospital, Yamaguchi University Hospital, Nagoya University Hospital, Okayama University Hospital, Kagawa University Hospital, Tokushima University Hospital, Kawasaki Medical School, Takasaki General Medical Center and Gunma Saiseikai Maebashi Hospital. Of the 837 patients, 796 were included in this study after excluding those with CP class C (*n* = 2), those without imaging evaluation (*n* = 27) and those with insufficient clinical data (*n* = 12). Of the 796 patients, 71 had CP‐B and 725 had CP‐A (Figure 1).

### 2.2 Diagnosis of HCC and Assessment of Liver Function

HCC was diagnosed based on biopsy or resection specimens obtained during the clinical course and/or characteristic radiological images consistent with the American Association for the Study of Liver Diseases criteria [8]. Tumour stage was assessed using the Barcelona Clinic Liver Cancer (BCLC) staging system. CP classification, albumin–bilirubin (ALBI) score and modified ALBI (mALBI) grade were used to evaluate the liver function [9, 10].

### 2.3 Treatment Protocol, Evaluation Criteria for Response and Follow‐Up of HCC

Intravenous treatment with 1200 mg atezolizumab and 15 mg/kg body weight bevacizumab was administered every 3 weeks. Three CP‐B cases included patients whose initial bevacizumab dose was reduced to 50% (7.5 mg/kg). Treatment was discontinued when undesirable occurrences or clinical tumour progression were observed. Treatment response was evaluated using contrast‐enhanced computed tomography or magnetic resonance imaging according to the Response Evaluation Criteria in Solid Tumours (RECIST) [11] every 8–12 weeks. Lesion measurements and treatment efficacy assessment were performed at each facility. The best response was adopted as the therapeutic effect.

### 2.4 Adverse Events

Adverse events (AEs) were assessed using the National Cancer Institute Common Terminology Criteria for Adverse Events (version 5.0) [12]. The incidence of immune‐related AEs (irAEs) and the need for corticosteroid treatment were also evaluated.

### 2.5 Ethical Considerations

Informed consent was obtained from all the participants on an opt‐out basis. The study protocol was approved by the Ethics Committee of Kurume University School of Medicine (approval code 23153) and conformed to the 1975 Declaration of Helsinki and Japanese Ethical Guidelines for Clinical Research (Ministry of Health, Labour and Welfare of Japan, Ethical Guidelines for Clinical Research, 2008).

### 2.6 Statistical Analysis

Continuous variables were dichotomized based on the median or clinically meaningful values in the multivariate analysis. Statistical analysis was performed using Wilcoxon's signed‐rank test and the Mann–Whitney U‐test. To estimate progression‐free survival (PFS) and overall survival (OS), we employed the Kaplan–Meier method and the log‐rank test. Cox proportional hazards regression analysis was performed to evaluate the risk factors for the survival of patients with HCC and CP‐B liver function. To prevent multicollinearity issues, highly correlated factors were combined, and the variance inflation factors of the final model variables were verified to be < 5. Variables with *p* values < 0.05 were selected and incorporated into the multiple regression model. Statistical significance was set at *p* < 0.05. Data analysis was performed using IBM SPSS Statistics ver. 22.0 (IBM Corp., Armonk, NY, USA).

## 3 Results

### 3.1 Patient Characteristics

The baseline characteristics of the 796 patients included in this study are summarised in Table 1. All patients were ICI treatment‐naïve. The median observation period was 14.6 months. Among CP‐B patients, 50/71 (70.4%) had a CP score (CPS) of 7. Compared with the CP‐A group, the CP‐B group had a significantly worse performance status (PS), larger tumour size and higher levels of tumour markers.

**TABLE 1:** Characteristics of the patients enrolled.

Variable		CP‐B (*n* = 71)	CP‐A (*n* = 725)	*p*
Age	Year	72.0 (64–79)	74.0 (68–80)	0.092
Sex	Male/female	56/15	590/135	0.606
BMI	kg/m^2^	23.60 (21.7–25.9)	23.20 (20.7–25.7)	0.272
Performance status	0 and 1	63 (88.7%)	700 (96.6%)	0.006
Child–Pugh score	7/8/9	50/19/2	—	—
ALBI	Score	−1.630 (−1.94 to −1.47)	−2.42 (−2.70 to −2.15)	< 0.001
mALBI grade	1/2a/2b/3	0/5/51/15	237/248/240/0	< 0.001
Tumour size	cm	4.50 (2.0–7.6)	3.1 (2.0–5.6)	0.033
Tumour number	≥ 7	26 (36.6%)	243 (33.6%)	0.196
Macroscopic PV invasion	Vp 3/4	12 (16.9%)	82 (11.3%)	0.163
Extrahepatic spread	Yes	22 (31.0%)	244 (33.7%)	0.649
BCLC stage	A/B/C	3/23/45	29/331/365	0.095
Aetiology	HBV/HCV/NBNC	6/23/42	92/263/370	0.157
Type 2 diabetes	Yes	33 (46.5%)	319 (44.0%)	0.688
Treatment line	First/later	48/23	512/213	0.595
ALT	U/mL	30.0 (21–47)	25.0 (18–40)	0.029
AFP	ng/mL	317.5 (10–2347)	25.0 (5–595)	0.001
DCP	mAU/mL	973.0 (144–16 640)	333.5 (53–3467)	0.003

*Note:* Data are presented as medians with interquartile range or numbers.

Abbreviations: AFP, alpha‐fetoprotein; ALBI, albumin–bilirubin; ALT, alanine aminotransferase; BCLC, Barcelona Clinic Liver Cancer; BMI, body mass index; CP‐A, Child–Pugh class A; CP‐B, Child–Pugh class B; DCP, des‐gamma‐carboxyprothrombin; HBV, hepatitis B virus; HCV, hepatitis C virus; mALBI, modified ALBI; NBNC, non‐B non‐C; PV, portal vein.

### 3.2 PFS, OS, and Response Rate Stratified by the CP Class

**FIGURE 2:**
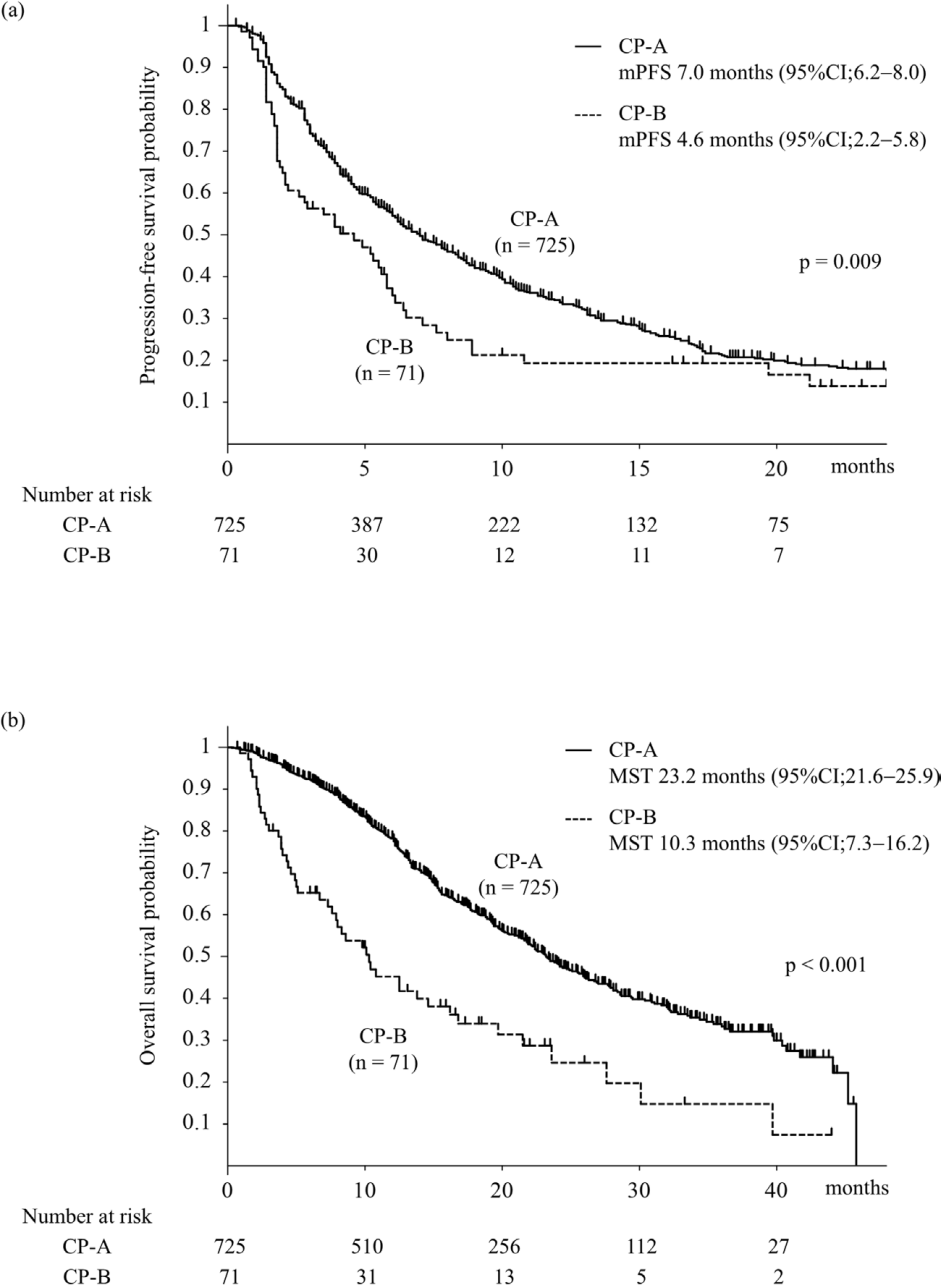
Progression‐free and overall survivals stratified by the Child–Pugh class. (a) Progression‐free survival in CP‐A versus CP‐B (median PFS, 7.0 vs. 4.6 months; *p* = 0.009). (b) Overall survival in CP‐A versus CP‐B (MST, 23.2 vs. 10.3 months; *p* < 0.001). CP‐A, Child–Pugh class A; CP‐A, Child–Pugh class A; CP‐B, Child–Pugh class B; CP‐B, Child–Pugh class B; CP‐C, Child–Pugh class C; HCC, hepatocellular carcinoma; MST, median survival time; PFS, progression‐free survival.

Figure 2 shows that PFS and OS were significantly shorter in CP‐B (median PFS, 4.6 months; median survival time [MST], 10.3 months) than in CP‐A (median PFS, 7.0 months; MST, 23.2 months) (PFS, *p* = 0.009; OS, *p* < 0.001). The efficacy of Atez/Bev in patients with unresectable HCC stratified by the CP class is shown in Table 2. In terms of the best response to Atez/Bev administration, the CP‐B group tended to have a higher progressive disease (PD) rate and a lower disease control (DC) rate than the CP‐A group. In the CP‐B group, the rate of transition to subsequent treatment was 37.1%, which is significantly lower than the 60.6% observed in the CP‐A group (*p* < 0.05).

**TABLE 2:** Response rate stratified by the Child–Pugh class.

Response category	CP‐B (*n* = 71)	CP‐A (*n* = 725)	*p*
CR	1 (1.4%)	22 (3.0%)	0.435
PR	16 (22.5%)	190 (26.2%)	0.500
SD	27 (38.0%)	361 (49.8%)	0.058
PD	27 (38.0%)	152 (21.0%)	0.001
OR	17 (23.9%)	212 (29.2%)	0.346
DC	44 (62.0%)	573 (79.0%)	0.001

Abbreviations: CP‐A, Child–Pugh class A; CP‐B, Child–Pugh class B; CR, complete response; DC, disease control; OR, objective response; PD, progressive disease; PR, partial response; SD, stable disease.

### 3.3 Frequency of AEs in the CP‐A and CP‐B Groups

The frequencies of AEs during Atez/Bev treatment in the CP‐A and CP‐B groups are shown in Table 3. Among grade ≥ 3 AEs, the incidence of bleeding‐related events was significantly higher in the CP‐B group than in the CP‐A group. However, when the analysis was limited to first‐line treatment, no significant differences in grade ≥ 3 AEs, including bleeding‐related events (CP‐A vs. CP‐B, 8.3% vs. 5.7%), were observed (Online resource 1). In addition, the discontinuation rate of Atez/Bev due to AEs was 30.7% for the CP‐A group and 36.9% for the CP‐B group, with no significant difference. The prevalence of corticosteroid use due to irAEs was 11.2% for the CP‐A group and 11.3% for the CP‐B group, with no significant difference.

**TABLE 3:** Frequency of adverse events.

	Any grade		Grade ≥ 3		*p*
	CP‐B (*n* = 71)	CP‐A (*n* = 725)	CP‐B (*n* = 71)	CP‐A (*n* = 725)	
All	52 (73.2%)	579 (79.9%)	23 (32.4%)	231 (31.9%)	0.926
Hypertension	17 (23.9%)	199 (27.4%)	3 (4.2%)	53 (7.3%)	0.332
Proteinuria	7 (9.8%)	194 (26.7%)	2 (2.8%)	78 (10.7%)	0.033
Elevated liver enzymes	23 (32.4%)	179 (24.7%)	5 (7.0%)	22 (3.0%)	0.075
Fever	9 (12.7%)	104 (14.3%)	1 (1.4%)	6 (0.8%)	0.616
Rash	5 (7.0%)	87 (12.0%)	0 (0.0%)	6 (0.8%)	0.441
Fatigue	8 (11.3%)	76 (10.5%)	1 (1.4%)	4 (0.6%)	0.383
Hypothyroidism	6 (8.4%)	62 (8.5%)	0 (0.0%)	4 (0.6%)	0.530
Bleeding‐related events	11 (15.5%)	60 (8.3%)	9 (12.7%)	34 (4.6%)	0.004
Diarrhoea	5 (7.0%)	59 (8.1%)	1 (1.4%)	6 (0.8%)	0.616
Decreased appetite	2 (2.8%)	48 (6.6%)	0 (0.0%)	4 (0.6%)	0.530
Oedema/ascites	5 (7.0%)	27 (3.7%)	1 (1.4%)	7 (1.0%)	0.721
Thrombocytopenia	3 (4.2%)	24 (3.3%)	1 (1.4%)	4 (0.6%)	0.383
Adrenocortical insufficiency	0 (0.0%)	20 (2.7%)	0 (0.0%)	10 (1.4%)	0.319
Interstitial pneumonia	1 (1.4%)	11 (1.5%)	1 (1.4%)	6 (0.8%)	0.616
Infusion reaction	1 (1.4%)	8 (1.1%)	0 (0.0%)	4 (0.6%)	0.530

Abbreviations: CP‐A, Child–Pugh class A; CP‐B, Child–Pugh class B.

### 3.4 PFS, OS, and Response Rate Stratified by the CPS and mALBI Grade

**FIGURE 3:**
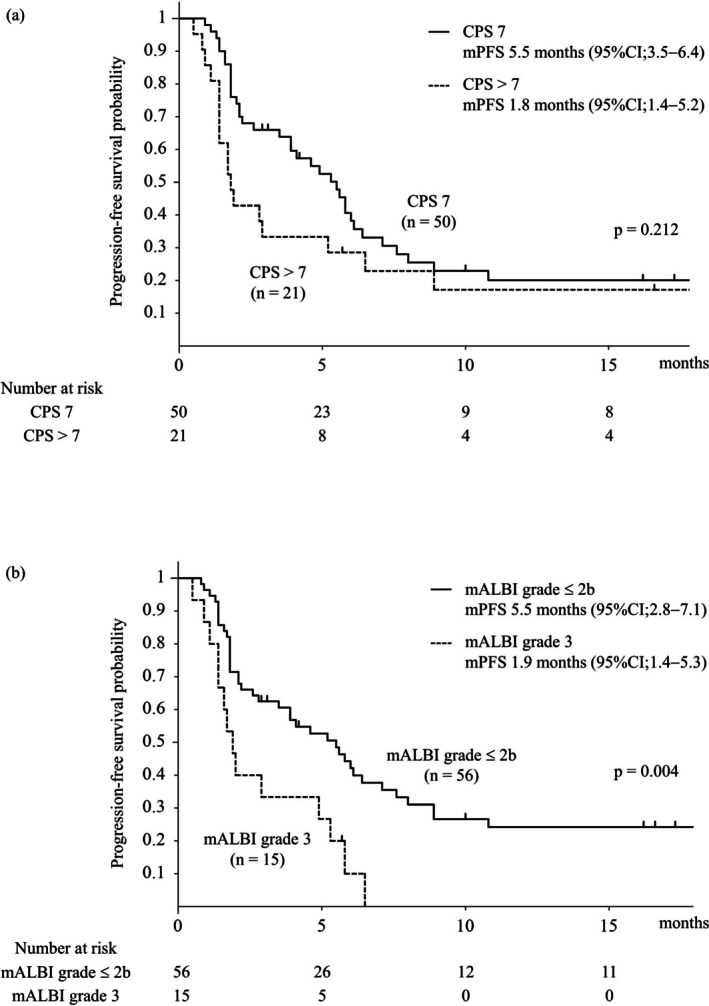
Progression‐free survival stratified by the Child–Pugh score and mALBI grade. (a) Progression‐free survival in CPS 7 versus CPS > 7 (median PFS, 5.5 vs. 1.8 months; *p* = 0.212). (b) Progression‐free survival in mALBI ≤ 2b versus grade 3 (median PFS, 5.5 vs. 1.9 months, *p* = 0.004). CPS, Child–Pugh score; mALBI, modified albumin–bilirubin; PFS, progression‐free survival.

**FIGURE 4:**
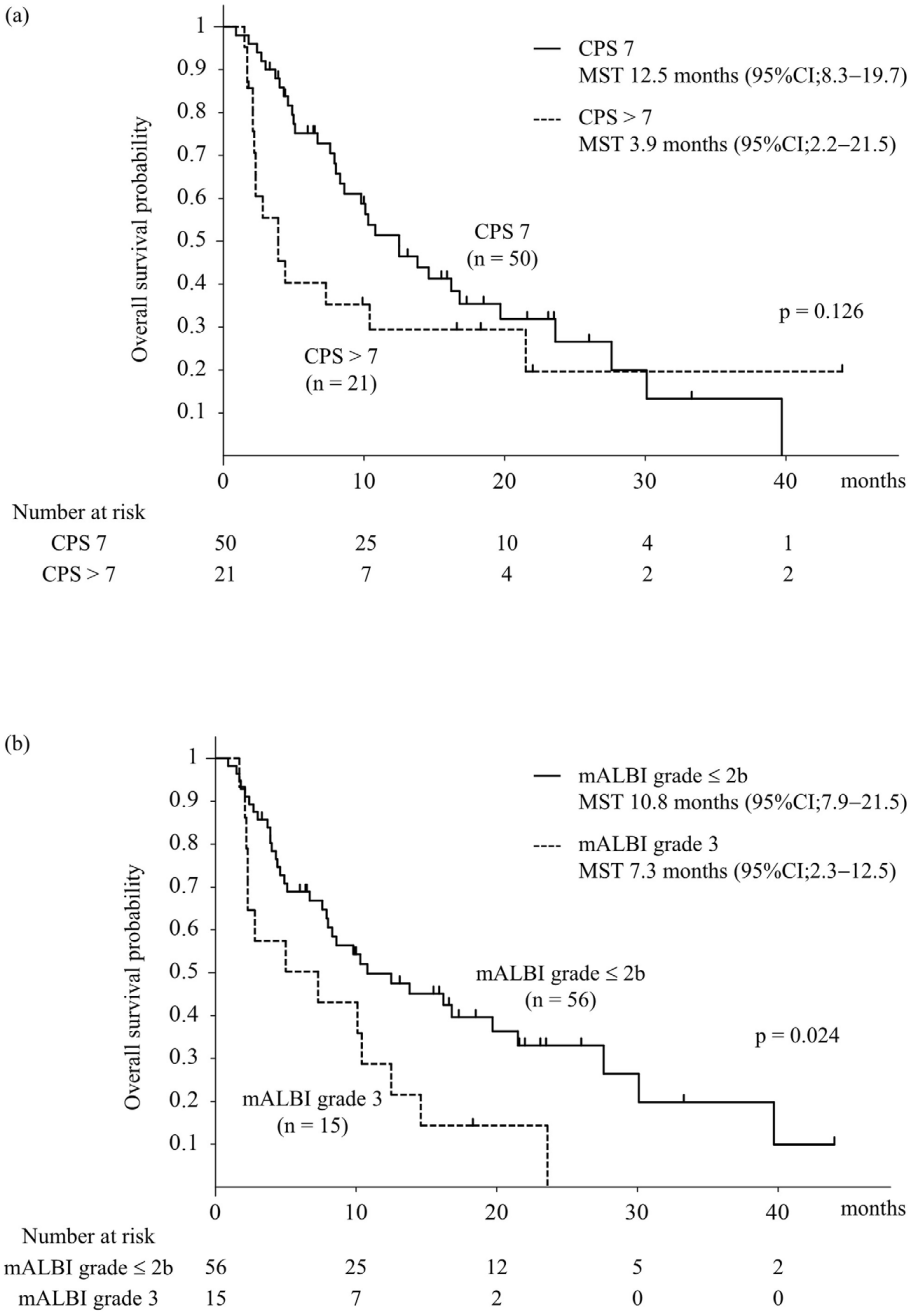
Overall survival stratified by the Child–Pugh score and mALBI grade. (a) Overall survival in CPS 7 versus CPS > 7 (MST, 12.5 vs. 3.9 months; *p* = 0.126). (b) Overall survival in mALBI ≤ 2b versus grade 3 (MST, 10.8 vs. 7.3 months; *p* = 0.024). CPS, Child–Pugh score; mALBI, modified albumin–bilirubin; MST, median survival time.

Figure 3 shows the PFS in patients with CP‐B liver function stratified by the CPS and mALBI grade. No significant differences in PFS were observed between the CPS 7 and CPS > 7 groups. However, significant differences in PFS were observed between the mALBI grade ≤ 2b and grade 3 groups (median PFS, 5.5 vs. 1.9 months; *p* = 0.004). Figure 4 shows the OS in patients with CP‐B liver function stratified by the CPS and mALBI grades. No significant differences in OS were observed between the CPS 7 and CPS > 7 groups. However, the OS differed significantly between the mALBI grade ≤ 2b and grade 3 groups (MST, 10.8 vs. 7.3 months; *p* = 0.024) (Online Resource 2). The baseline characteristics of patients with CP‐B liver function stratified according to the mALBI grade are summarised in Online Resource 3. No significant difference in background factors was observed between the mALBI grade ≤ 2b and grade 3 groups. The response rates of Atez/Bev stratified by the mALBI grade for patients with CP‐B liver function are shown in Online Resource 4. Patients with mALBI grade 3 had significantly higher PD rates and lower DC rates than those with mALBI grade ≤ 2b (*p* = 0.001). Furthermore, even when limited to the 48 first‐line therapy in the CP‐B group, a significant difference in PFS and OS was observed with stratification using mALBI grading (Online Resource 5).

### 3.5 Univariate and Multivariate Analyses of Factors Contributing to OS in Patients With CP‐B Liver Function Treated With Atez/Bev for HCC

In the univariate analysis, mALBI grade 3, tumour size > 4.5 cm, BCLC stage C, treatment line and alpha‐fetoprotein level > 317.5 ng/mL were identified as factors contributing to OS. In multivariate analysis, the mALBI grade (hazard ratio [95% CI]: 2.388 [1.186–4.810], *p* = 0.014) was extracted as a factor contributing to OS (Table 4).

**TABLE 4:** Multivariate analysis of factors contributing to overall survival using the Cox proportional hazards model.

Factor		Univariate analysis		Multivariate analysis	
		HR (95% CI)	*p*	HR (95% CI)	*p*
Age	> 72 years	1.437 (0.809–2.552)	0.215		
Sex	Male	0.901 (0.434–1.869)	0.779		
BMI	> 23.6 kg/m^2^	0.764 (0.431–1.354)	0.357		
Performance status	> 1	1.978 (0.854–4.578)	0.111		
Child–Pugh score	> 7	1.651 (0.872–2.999)	0.126		
mALBI grade	3	2.082 (1.089–3.982)	0.026	2.058 (1.048–4.042)	0.036
Tumour number	≥ 7	1.674 (0.940–2.982)	0.079		
BCLC stage	C	2.020 (1.170–3.488)	0.011	1.543 (0.839–2.837)	0.162
Aetiology	NBNC	1.194 (0.661–2.158)	0.555		
Type 2 diabetes	Yes	1.294 (0.726–2.305)	0.381		
Treatment line	Later	1.861 (1.028–3.367)	0.040	1.519 (0.832–2.773)	0.173
ALT	> 30 IU/mL	1.389 (0.778–2.480)	0.266		
AFP	> 317.5 ng/mL	2.020 (1.134–3.600)	0.016	1.725 (0.896–3.322)	0.102
DCP	> 973 mAU/mL	1.272 (0.668–2.442)	0.462		

Abbreviations: AFP, alpha‐fetoprotein; ALT, alanine aminotransferase; BCLC, Barcelona Clinic Liver Cancer; BMI, body mass index; CI, confidence interval; DCP, des‐gamma‐carboxy prothrombin; HR, hazard ratio; mALBI, modified albumin–bilirubin; NBNC, non‐B non‐C.

## 4 Discussion

Systemic therapy for patients with unresectable HCC and poor hepatic reserve is a major challenge in clinical practice. Although several reports have been published on systemic therapy for patients with HCC and CP‐B liver function [13, 14, 15], there is currently no evidence to demonstrate the efficacy and safety of this approach. It was unclear which patients with CP‐B liver function would benefit from the current first‐line Atez/Bev therapy. This retrospective study evaluated the conditions under which Atez/Bev therapy is recommended for patients with CP‐B liver function.

First, the safety profile of Atez/Bev in CP‐B did not differ from that in CP‐A. The discontinuation rates due to AEs and the proportion of patients with grade ≥ 3AEs were similar between the CP‐B and CP‐A groups. Late‐line treatment in the CP‐B group was associated with a higher frequency of variceal rupture. As a result, grade 3 or higher bleeding‐related events occurred significantly more often in the CP‐B group than in the CP‐A group overall; however, no significant difference was observed when the analysis was limited to first‐line treatment. There is no consensus regarding the reported safety profiles of CP‐B and CP‐A, with some reports indicating similar incidences of AEs [16, 17], whereas others suggest that CP‐B is more likely to cause serious AEs [18, 19]. In our study, the frequency of AEs was similar; however, the administration period was more extended for CP‐A, which may have had an impact on bevacizumab‐related AEs, particularly proteinuria. However, our study population included patients with suboptimal CP‐B conditions, such as poor PS, large tumour size and high tumour marker levels, in addition to an impaired hepatic reserve. Considering the unfavourable clinical manifestations associated with CP‐B, the rates of serious AEs and discontinuations due to AEs were similar, demonstrating that the safety profile of CP‐B is well tolerated in clinical practice. Nevertheless, caution remains warranted for bleeding‐related events, especially during late‐line treatment.

Second, the Atez/Bev treatment outcomes of patients with CP‐B liver function cases can be further stratified by the mALBI grade. For patients with HCC and CP‐B liver function, the CPS did not allow for a clear stratification of treatment outcomes. However, classification by the mALBI grade enabled a clear stratification of the response rate, PFS and OS. Even in CP‐B, in patients whose hepatic reserve function was maintained within mALBI grade 2b, the objective response rate was 28.6%, the median PFS was 5.5 months, and the MST was 10.8 months, suggesting that Atez/Bev therapy may be effective. There have been several reports on the outcomes (MST, 5.8–13.4 months) of Atez/Bev therapy in patients with CP‐B liver function [14, 15, 16, 17, 18, 20], and the results of this study were comparable to those previously reported. Our results suggest that Atez/Bev administration to patients with CP‐B liver function may be clinically effective in patients with mALBI grade ≤ 2b. Furthermore, patients with mALBI grade 3 had a poor prognosis (median PFS, 1.9 months; MST, 7.3 months), which was comparable to previously reported results for the best supportive care (BSC) [13]. While the BSC may be an option for patients with a low hepatic reserve, Fulgenzi et al. reported that ICI regimens are effective against BSC. The outcomes of administering Atez/Bev to patients with mALBI grade 3 were poor, and further investigation is needed to determine whether there is an advantage in choosing ICI‐based regimen therapy in these patients with poor hepatic reserve.

While this study only examined Atez/Bev therapy, vascular endothelial growth factor (VEGF) inhibitors may also affect hepatic reserve function. Previous studies reported an MST of 5.2 months with sorafenib [21] and 8.8 months with lenvatinib [14] in Child–Pugh class B patients undergoing treatment with molecular target agents. However, a direct comparison with the findings of the present study is challenging due to differences in subsequent treatment options available during different time periods. Other ICI regimens that do not contain VEGF inhibitors may have a less significant impact on hepatic reserve function. However, it is essential to note that fewer serious AEs required administration of corticosteroids in addition to Atez/Bev therapy in both the CP‐A (11.2%) and CP‐B (11.3%) groups. We speculate that the low incidence of serious irAEs may have contributed to a lower rate of Atez/Bev discontinuation due to AEs in the CP‐B group, even in patients without a maintained hepatic reserve. VEGF‐free regimens using anti‐CTLA‐4 plus PD‐1/PD‐L1 have a higher rate of corticosteroid requirement due to irAEs compared with PD‐1/PD‐L1 monotherapy [5, 22, 23], and it is predicted that CP‐B has a higher risk of discontinuation due to irAEs. The optimal ICI regimen for patients with CP‐B liver function is a topic of importance for future studies.

One of the limitations of the present study is its retrospective nature. Additionally, the treatment effects and AEs were not centrally assessed; therefore, there may be heterogeneity depending on the judgement of each institution. Another limitation is the lack of a control group. This study did not assess whether Atez/Bev was superior to molecular targeted agents and other ICI‐based regimens. In addition, an initial dose adjustment was made in three CP‐B cases, but the small sample size precluded a robust evaluation of its clinical impact on the efficacy and safety of Atezo/Bev in these patients. Future studies are needed to verify the efficacy of Atez/Bev in patients with CP‐B liver function.

In conclusion, Atez/Bev therapy is expected to have a certain degree of efficacy and safety in patients with CP‐B liver function, especially if their hepatic reserve is maintained within mALBI grade 2b.

## Author Contributions

Conceptualization: Ryu Sasaki; Methodology: Ryu Sasaki; Formal analysis and investigation: Ryu Sasaki; Data curation: Ryu Sasaki, Shigeo Shimose, Issei Saeki, Takanori Ito, Yasuto Takeuchi, Joji Tani, Tetsu Tomonari, Kyo Sasaki, Satoru Kakizaki, Takeshi Hatanaka, Satoshi Miuma, Tomotake Shirono, Hideki Iwamoto, Norikazu Tanabe, Takafumi Yamamoto, Yuki Kanayama, Atsushi Naganuma; Writing – original draft: Ryu Sasaki; Writing – review and editing: Ryu Sasaki, Shigeo Shimose, Issei Saeki, Takanori Ito, Yasuto Takeuchi, Joji Tani, Tetsu Tomonari, Kyo Sasaki, Satoru Kakizaki, Takeshi Hatanaka, Sohji Nishina, Tetsuji Takayama, Hideki Kobara, Motoyuki Otsuka, Hiroki Kawashima, Taro Takami, Takumi Kawaguchi, Hisamitsu Miyaaki; Funding acquisition: Ryu Sasaki; Visualisation: Ryu Sasaki; Validation: Ryu Sasaki; Software: Ryu Sasaki; Project administration: Ryu Sasaki, Shigeo Shimose; Resources: Ryu Sasaki; Supervision: Shigeo Shimose, Issei Saeki, Takanori Ito, Sohji Nishina, Tetsuji Takayama, Hideki Kobara, Motoyuki Otsuka, Hiroki Kawashima, Taro Takami, Takumi Kawaguchi, Hisamitsu Miyaaki.

## Funding

The authors have nothing to report.

## Conflicts of Interest

Shigeo Shimose received lecture fees from AstraZeneca, Eisai and Chugai Pharmaceutical Co. Ltd. Issei Saeki received lecture fees from AstraZeneca. Takanori Ito received lecture fees from Chugai Pharmaceutical Co. Ltd. and AstraZeneca and grants from Chugai Pharmaceutical Co. Ltd. Satoru Kakizaki received lecture fees from AbbVie GK. Hiroki Kawashima received lecture fees from AstraZeneca and grants from Chugai Pharmaceutical Co. Ltd. Taro Takami received lecture fees from AstraZeneca and Chugai Pharmaceutical Co. Ltd. and grants from Chugai Pharmaceutical Co. Ltd. Takumi Kawaguchi received lecture fees from ASKA Pharmaceutical Co. Ltd., Taisho Pharmaceutical Co. Ltd., Kowa Company Ltd., AbbVie GK., Eisai Co. Ltd., EA Pharma Co. Ltd., Nippon Boehringer Ingelheim Co. Ltd., Sumitomo Pharma Co. Ltd., Novo Nordisk Pharma Ltd., Otsuka Pharmaceutical Co. Ltd. and Janssen Pharmaceutical K.K. The other authors declare no conflicts of interest.

## Supporting information


**Online Resource 1.** Subgroup analysis of the first‐line therapy (adverse events).

**Online Resource 2.** Characteristics of the patients with Child–Pugh class B liver function stratified by the mALBI grade.

**Online Resource 3.** Response rate stratified by the mALBI grade.

**Online Resource 4.** Progression‐free and overall survival stratified by the mALBI grade. (a) Progression‐free survival in mALBI 2a versus 2b versus 3 (median PFS, NA vs. 5.2 months vs. 1.9 months). (b) Overall survival in mALBI 2a versus 2b versus 3 (MST, 39.7 months vs. 10.3 months vs. 7.3 months). mALBI, modified albumin–bilirubin; MST, median survival time; PFS, progression‐free survival.

**Online Resource 5.** Subgroup analysis of the first‐line therapy in the CP‐B group: progression‐free and overall survival stratified by the Child–Pugh score and mALBI grade. (a) Progression‐free survival in CPS 7 versus CPS > 7 (median PFS, 5.6 months vs. 1.8 months; *p* = 0.690). (b) Progression‐free survival in mALBI ≤ 2b versus mALBI 3 (median PFS, 5.8 months vs. 1.6 months; *p* = 0.007). (c) Overall survival in CPS 7 versus CPS > 7 (MST, 13.8 months vs. 3.9 months; *p* = 0.536). (d) Overall survival in mALBI ≤ 2b versus mALBI 3 (MST, 16.2 months vs. 5.0 months; *p* = 0.014). CPS, Child–Pugh score; mALBI, modified albumin–bilirubin; MST, median survival time; PFS, progression‐free survival.

## Data Availability

The data that support the findings of this study are available on request from the corresponding author. The data are not publicly available due to privacy or ethical restrictions.
