# Supplementary material for: Efficacy and Safety of Atezolizumab Plus Bevacizumab for Patients With Hepatocellular Carcinoma and Child–Pugh Class B
Source: Liver Int. 2025 Nov 28;46(1):e70466. doi: 10.1111/liv.70466 (PMC12661480; doi:10.1111/liv.70466)
Supplement: Supplementary file 1 — Online Resource 1. Subgroup analysis of the first‐line therapy (adverse events). [file LIV-46-0-s002.docx]

| Online Resource 1. Subgroup analysis of the first-line therapy (Adverse events) | | | | | | |
| --- | --- | --- | --- | --- | --- | --- |
|  | Any grade | |  | Grade ≥ 3 | | p-value |
|  | CP-B (n = 48) | CP-A (n = 512) |  | CP-B (n = 48) | CP-A (n = 512) |  |
| All | 32 (66.7%) | 403 (78.7%) |  | 13 (27.1%) | 163 (31.8%) | 0.497 |
| Hypertension | 11 (22.9%) | 148 (28.9%) |  | 2 (4.2%) | 43 (8.4%) | 0.302 |
| Proteinuria | 4 (8.3%) | 115 (22.4%) |  | 1 (2.1%) | 45 (8.8%) | 0.105 |
| Elevated liver enzymes | 14 (29.1%) | 115 (22.4%) |  | 3 (6.3%) | 18 (3.5%) | 0.340 |
| Fever | 4 (8.3%) | 75 (14.6%) |  | 1 (2.1%) | 4 (0.8%) | 0.359 |
| Rash | 5 (10.5%) | 56 (10.9%) |  | 0 (0.0%) | 5 (1.0%) | 0.491 |
| Fatigue | 6 (12.5%) | 4 (0.8%) |  | 1 (2.1%) | 4 (0.8%) | 0.359 |
| Hypothyroidism | 3 (6.3%) | 44 (8.6%) |  | 0 (0.0%) | 4 (0.8%) | 0.538 |
| Bleeding-related events | 5 (10.4%) | 46 (9.0%) |  | 4 (8.3%) | 29 (5.7%) | 0.452 |
| Diarrhea | 4 (8.3%) | 42 (8.2%) |  | 0 (0.0%) | 4 (0.8%) | 0.538 |
| Decreased appetite | 1 (2.1%) | 31 (6.1%) |  | 0 (0.0%) | 2 (0.4%) | 0.664 |
| Edema/ascites | 2 (4.2%) | 22 (4.3%) |  | 0 (0.0%) | 6 (1.2%) | 0.450 |
| Thrombocytopenia | 3 (6.3%) | 17 (3.3%) |  | 1 (2.1%) | 2 (0.4%) | 0.124 |
| Adrenocortical insufficiency | 0 (0.0%) | 14 (2.7%) |  | 0 (0.0%) | 6 (1.2%) | 0.450 |
| Interstitial pneumonia | 0 (0.0%) | 9 (1.8%) |  | 0 (0.0%) | 5 (0.9%) | 0.491 |
| Infusion reaction | 1 (2.1%) | 4 (0.8%) |  | 0 (0.0%) | 3 (0.6%) | 0.594 |

CP-B, Child–Pugh class B; CP-A, Child–Pugh class A
