# Supplementary material for: Efficacy and Safety of Atezolizumab Plus Bevacizumab for Patients With Hepatocellular Carcinoma and Child–Pugh Class B
Source: Liver Int. 2025 Nov 28;46(1):e70466. doi: 10.1111/liv.70466 (PMC12661480; doi:10.1111/liv.70466)
Supplement: Supplementary file 2 — Online Resource 2. Characteristics of the patients with Child–Pugh class B liver function stratified by the mALBI grade. [file LIV-46-0-s004.docx]

| Online Resource 2. Characteristics of the patients with Child–Pugh class B liver function stratified by the mALBI grade | | | |  |
| --- | --- | --- | --- | --- |
|  |  |  |  |  |
| Variable |  | mALBI ≤ 2b (n = 56) | mALBI 3 (n = 15) | p-value |
| Age | year | 71.5 (64−79) | 75.0 (63−78) | 0.761 |
| Sex | male/female | 44/12 | 12/3 | 0.904 |
| BMI | kg/m^2^ | 23.30 (21.7−25.9) | 24.10 (21.6−25.9) | 0.805 |
| Performance status | 0/1 | 50 (89.3%) | 13 (86.7%) | 0.673 |
| Tumor size | cm | 4.50 (2.0−8.3) | 4.3 (2.4−6.5) | 0.943 |
| Tumor number | ≥ 7 | 20 (35.7%) | 6 (40.0%) | 0.674 |
| Macroscopic PV invasion | Vp 3/4 | 10 (17.9%) | 2 (13.3%) | 0.677 |
| Extrahepatic spread | yes | 12 (26.8%) | 7 (46.7%) | 0.207 |
| BCLC stage | A/B/C | 3/20/33 | 0/3/12 | 0.281 |
| Etiology | HBV/HCV/NBNC | 4/17/35 | 2/6/7 | 0.247 |
| Type 2 diabetes | yes | 27 (48.2%) | 6 (40.0%) | 0.571 |
| Treatment line | 1st/later | 39/17 | 9/6 | 0.540 |
| ALT | U/mL | 30.0 (20−49) | 28.0 (22−42) | 0.810 |
| AFP | ng/mL | 439.5 (10−2,284) | 45.0 (11−2,517) | 0.730 |
| DCP | mAU/mL | 580.0 (125−13,097) | 6,338.0 (429−27,968) | 0.089 |

Data are given as the medians with interquartile range or numbers.

BMI, body mass index; ALBI, albumin–bilirubin; mALBI, modified ALBI; PV, portal vein; BCLC, Barcelona Clinic Liver Cancer; HBV, hepatitis B virus; HCV, hepatitis C virus; NBNC, non-B non-C; ALT, alanine aminotransferase; AFP, alpha-fetoprotein; DCP, des-gamma-carboxyprothrombin
