# Supplementary material for: Efficacy and Safety of Atezolizumab Plus Bevacizumab for Patients With Hepatocellular Carcinoma and Child–Pugh Class B
Source: Liver Int. 2025 Nov 28;46(1):e70466. doi: 10.1111/liv.70466 (PMC12661480; doi:10.1111/liv.70466)
Supplement: Supplementary file 3 — Online Resource 3. Response rate stratified by the mALBI grade. [file LIV-46-0-s003.docx]

| Online Resource 3. Response rate stratified by the mALBI grade | | |  |
| --- | --- | --- | --- |
|  |  |  |  |
| Response category | mALBI ≤ 2b (n = 56) | mALBI 3 (n = 15) | p-value |
| CR | 1 (1.8%) | 0 (0.0%) | 0.602 |
| PR | 15 (26.8%) | 1 (6.7%) | 0.097 |
| SD | 23 (41.1%) | 4 (26.7) | 0.307 |
| PD | 17 (30.4%) | 10 (66.7%) | 0.010 |
| OR | 16 (28.6%) | 1 (6.7%) | 0.077 |
| DC | 39 (69.6%) | 5 (33.3%) | 0.010 |

mALBI, modified albumin–bilirubin; CR, complete response; PR, partial response; SD, stable disease; PD, progressive disease; OR, objective response; DC, disease control
