# Supplementary figures and images for: Efficacy and Safety of Atezolizumab Plus Bevacizumab for Patients With Hepatocellular Carcinoma and Child–Pugh Class B
Source: Liver Int. 2025 Nov 28;46(1):e70466. doi: 10.1111/liv.70466 (PMC12661480; doi:10.1111/liv.70466)

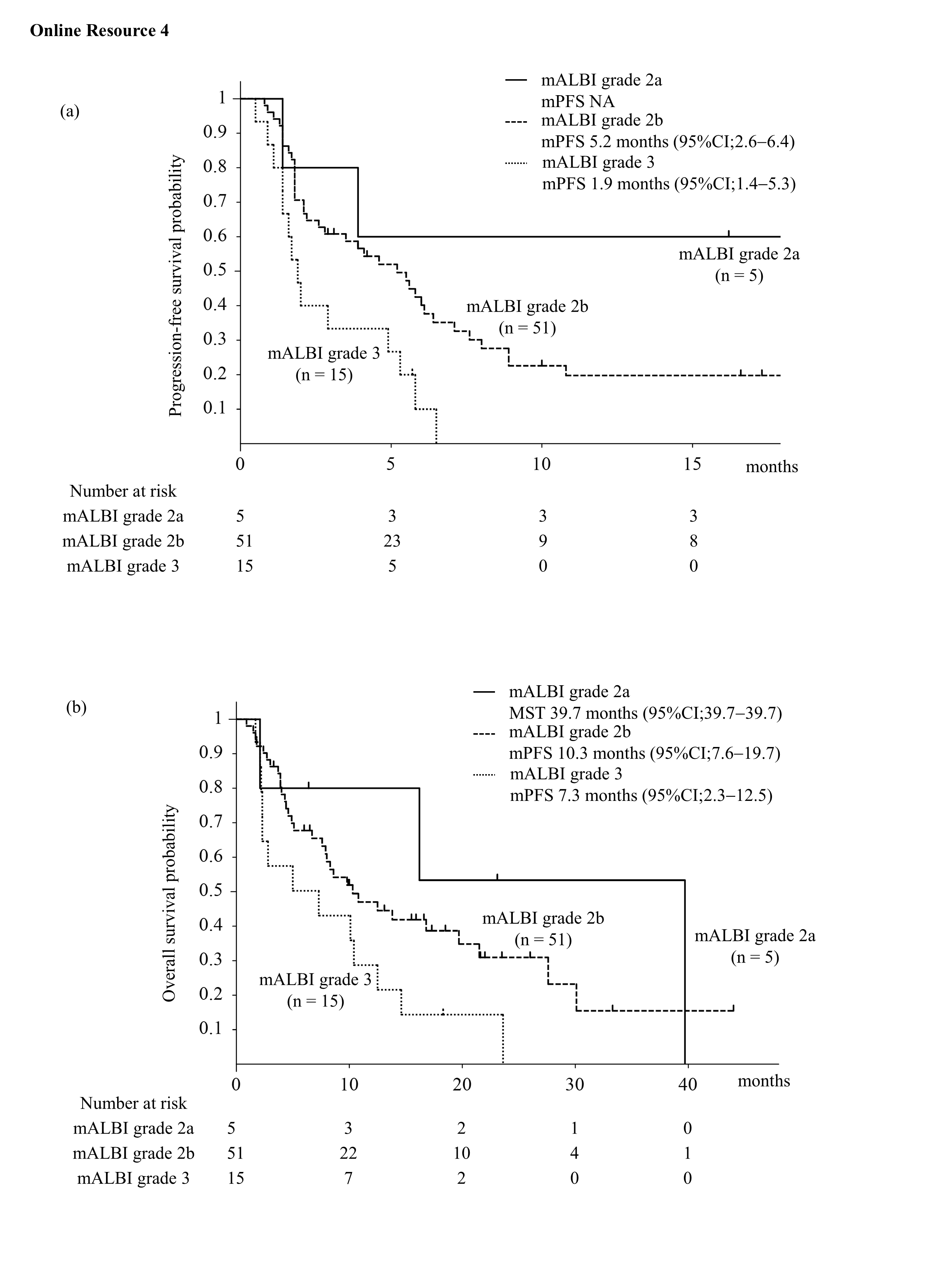

Supplement: Supplementary file 4 — Online Resource 4. Progression‐free and overall survival stratified by the mALBI grade. (a) Progression‐free survival in mALBI 2a versus 2b versus 3 (median PFS, NA vs. 5.2 months vs. 1.9 months). (b) Overall survival in mALBI 2a versus 2b versus 3 (MST, 39.7 months vs. 10.3 months vs. 7.3 months). mALBI, modified albumin–bilirubin; MST, median survival time; PFS, progression‐free survival. [file LIV-46-0-s001.tif]

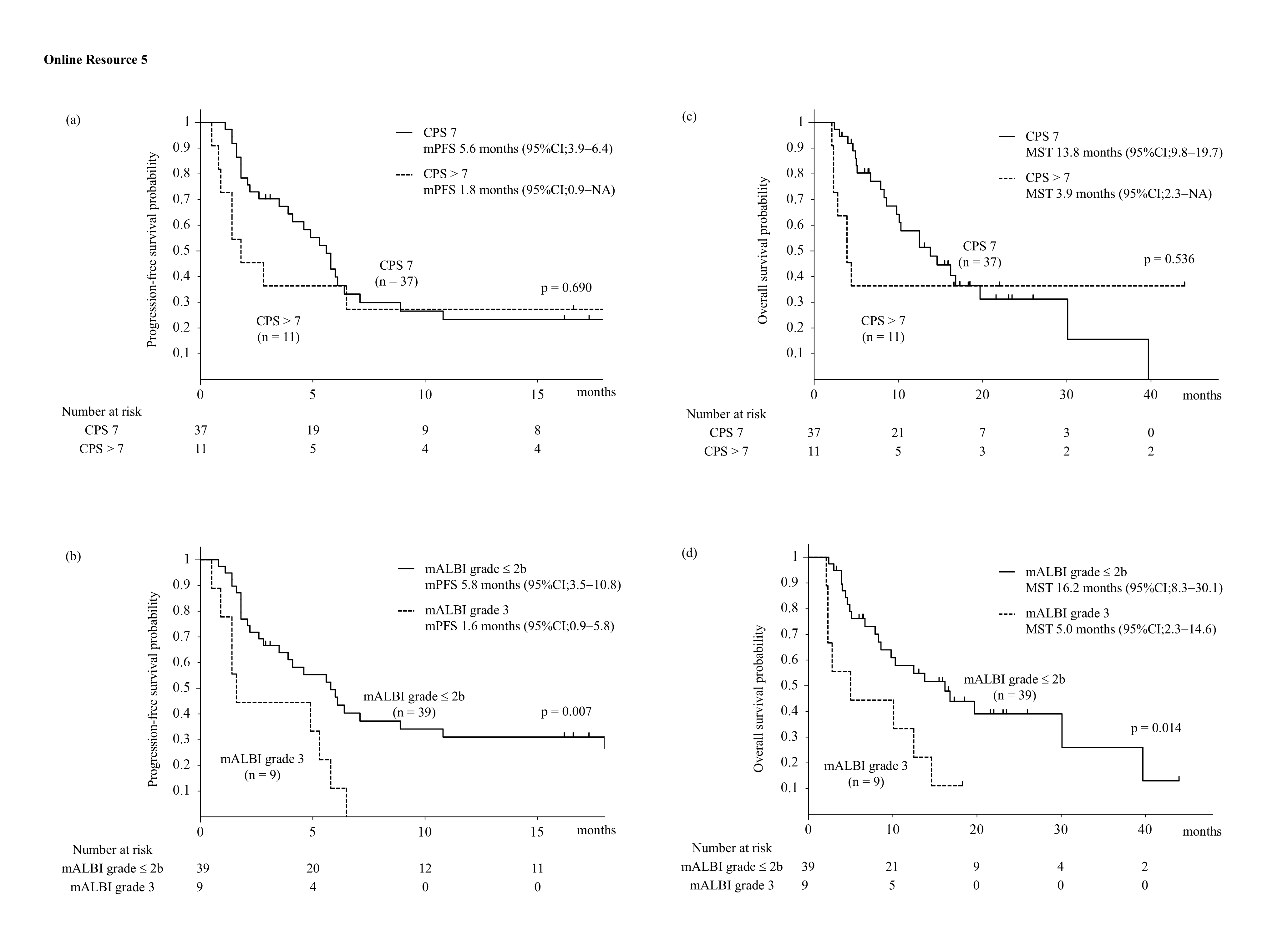

Supplement: Supplementary file 5 — Online Resource 5. Subgroup analysis of the first‐line therapy in the CP‐B group: progression‐free and overall survival stratified by the Child–Pugh score and mALBI grade. (a) Progression‐free survival in CPS 7 versus CPS > 7 (median PFS, 5.6 months vs. 1.8 months; p = 0.690). (b) Progression‐free survival in mALBI ≤ 2b versus mALBI 3 (median PFS, 5.8 months vs. 1.6 months; p = 0.007). (c) Overall survival in CPS 7 versus CPS > 7 (MST, 13.8 months vs. 3.9 months; p = 0.536). (d) Overall survival in mALBI ≤ 2b versus mALBI 3 (MST, 16.2 months vs. 5.0 months; p = 0.014). CPS, Child–Pugh score; mALBI, modified albumin–bilirubin; MST, median survival time; PFS, progression‐free survival. [file LIV-46-0-s005.tif]
